# Actin clearance promotes polarized dynein accumulation at the immunological synapse

**DOI:** 10.1371/journal.pone.0210377

**Published:** 2019-07-03

**Authors:** Elisa Sanchez, Xin Liu, Morgan Huse

**Affiliations:** Immunology Program, Memorial Sloan-Kettering Cancer Center, New York, NY, United States of America; University of Iowa, UNITED STATES

## Abstract

Immunological synapse (IS) formation between a T cell and an antigen-presenting cell is accompanied by the reorientation of the T cell centrosome toward the interface. This polarization response is thought to enhance the specificity of T cell effector function by enabling the directional secretion of cytokines and cytotoxic factors toward the antigen-presenting cell. Centrosome reorientation is controlled by polarized signaling through diacylglycerol (DAG) and protein kinase C (PKC). This drives the recruitment of the motor protein dynein to the IS, where it pulls on microtubules to reorient the centrosome. Here, we used T cell receptor photoactivation and imaging methodology to investigate the mechanisms controlling dynein accumulation at the synapse. Our results revealed a remarkable spatiotemporal correlation between dynein recruitment to the synaptic membrane and the depletion of cortical filamentous actin (F-actin) from the same region, suggesting that the two events were causally related. Consistent with this hypothesis, we found that pharmacological disruption of F-actin dynamics in T cells impaired both dynein accumulation and centrosome reorientation. DAG and PKC signaling were necessary for synaptic F-actin clearance and dynein accumulation, while calcium signaling and microtubules were dispensable for both responses. Taken together, these data provide mechanistic insight into the polarization of cytoskeletal regulators and highlight the close coordination between microtubule and F-actin architecture at the IS.

## Introduction

Antigen recognition by the T cell receptor (TCR) induces the formation of a stereotyped interface between the T cell and the antigen-presenting cell known as the immunological synapse (IS) [1]. The IS maintains strong adhesion, regulates TCR signaling, and enables polarized intercellular communication. Critical to IS assembly is the reorientation of the T cell centrosome (also called the microtubule organizing center, or MTOC) to the center of the interface [2, 3]. The centrosome polarizes to the IS within minutes of initial TCR stimulation, carrying with it the Golgi apparatus, endosomal compartment, and, in cytotoxic T cells, lytic granules containing perforin and granzyme. This is though to promote the directional secretion of cytokines and cytotoxic factors toward the APC, thereby enhancing the specificity of these critical effector responses.

The centrosome is guided to the IS by cytoskeletal regulators that push and pull on the microtubule cytoskeleton [4–7]. Prior studies indicate that dynein, the predominant minus end-directed microtubule motor in eukaryotes, is particularly important for this process. Dynein accumulates at the IS shortly after TCR stimulation, and depletion or inhibition of the protein strongly impairs centrosome reorientation [5–9]. These results are consistent with a model in which dynein, after it becomes anchored at the synaptic membrane, pulls on microtubules to reposition the centrosome. How TCR signaling events modulate the localization of this molecular motor remains an area of active study. It is known that recruitment of dynein, and ultimately the centrosome, depends on the polarized accumulation of the lipid second messenger diacylglycerol (DAG) [8], which is generated via the hydrolysis of phosphatidylinostitol-4,5-bisphosphate (PIP_2_) by phospholipase C-γ (PLC-γ) downstream of the TCR. DAG forms a gradient centered at the IS, which drives cytoskeletal polarization, at least in part, by recruiting and activating members of the novel protein kinase C (nPKC) subfamily [10]. Precisely how DAG and nPKC signaling promote recruitment of dynein, however, is poorly understood.

During IS assembly, cortical F-actin reorganizes into an annular configuration characterized by intense actin polymerization at the periphery of the contact and clearance from the center [1]. As with centrosome reorientation, formation of the synaptic F-actin ring depends strongly on phosphoinositide signaling. PIP_2_ depletion by PLC-γ promotes F-actin depletion at the center of the IS, while PIP_2_ phosphorylation by phosphoinositide 3-kinase drives F-actin growth in the periphery [11, 12]. Furthermore, centrosome reorientation and F-actin ring formation are tightly coupled in time, with the centrosome moving to the IS just as F-actin clears from the central synaptic membrane [13]. These molecular and temporal relationships imply a close, and perhaps causal, link between F-actin remodeling and centrosome recruitment. Indeed, perturbations that disrupt F-actin ring formation also impair the centrosome polarization response [11, 14]. Whether synaptic F-actin dynamics influence dynein localization, however, has not been explored.

In the present study, we applied a TCR photoactivation and imaging approach to investigate the mechanism of dynein localization to the IS. We found that F-actin depletion from the synaptic membrane was closely correlated with dynein accumulation in space and time, and that pharmacological inhibition of F-actin dynamics prevented both dynein recruitment and centrosome reorientation. These polarization responses were dependent on both DAG and PKC signaling, but did not require dynein activity or elevated cytoplasmic calcium (Ca^2+^). Taken together, these data establish the molecular and cellular framework for dynein polarization in T cells and provide insight into the assembly of immune cell-cell interactions.

## Results

### Localized TCR stimulation induces the recruitment of dynein 1 complexes

To study the recruitment dynamics of dynein in the context of polarized T cell activation, we employed an imaging-based method that enables spatiotemporally controlled stimulation of the TCR [15, 16]. In this system, CD4^+^ T cells expressing the 5C.C7 TCR are added to glass surfaces coated with a photoactivatable form of their cognate ligand, the moth cytochrome c_88-103_ (MCC) peptide bound to the class II MHC protein I-E^k^ (MCC-I-E^k^). This photoactivatable peptide-MHC complex (called NPE-MCC-I-E^k^) is nonstimulatory to the 5C.C7 TCR until it is irradiated with ultraviolet (UV) light. After the T cells attach to the surface, focused UV irradiation is used to generate a micron-sized region of cognate peptide-MHC beneath an individual T cell, inducing localized TCR signaling responses and cytoskeletal remodeling events that can be monitored by epifluorescence or total internal reflection fluorescence (TIRF) microscopy. Using this system, we have found that TCR stimulation drives DAG gradient formation and the ordered recruitment of nPKC isoforms within 90 seconds [10]. This is followed by the localized accumulation of dynein ~10 seconds later and the reorientation of the centrosome ~5 seconds after that [8].

**Fig 1:**
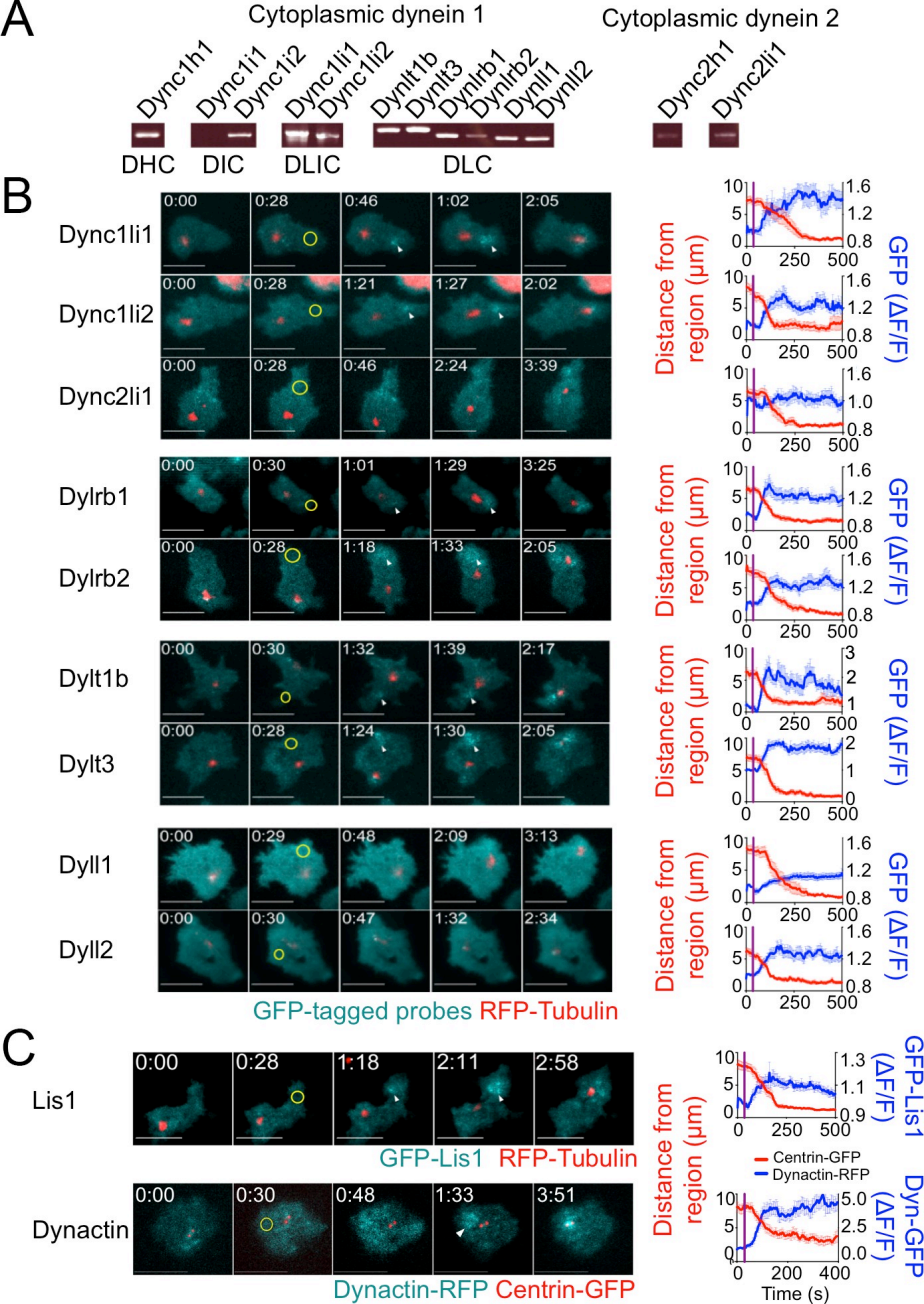
Cytoplasmic dynein 1 is recruited to the site of TCR stimulation. (A) RT-PCR analysis of the indicated dynein subunits, using RNA derived from 5C.C7 T cell blasts. (B-C) TCR photoactivation experiments were performed using 5C.C7 T cell blasts expressing fluorescently labeled tubulin or centrin (to visualize the centrosome) together with fluorescent probes for the indicated dynein subunits (B) or cofactors (C). Left, representative time-lapse montages showing TIRF images of the indicated dynein subunit or dynein cofactor and epifluorescence images of the centrosome. In each experiment, the moment and location of UV irradiation is indicated by a yellow circle. White arrowheads denote dynein or dynein cofactor accumulation in the plasma membrane prior to centrosome recruitment. Time in M:SS is indicated in the top left corner of each image. Right, graphs documenting the average dynein or dynein cofactor accumulation response (blue) and centrosome reorientation (red) in each data set. Error bars denote standard error of the mean (SEM). The moment of UV irradiation is indicated in each graph by a vertical purple line. Scale bars = 10 μm. In B, N ≥ 8 cells for each condition. In C, N ≥ 10 cells for each condition.

The precise subunit composition of cytoplasmic dynein varies depending on the cell type and intracellular compartment in question [17]. Using RT-PCR, we found that murine T cells express the full complement of cytoplasmic dynein 1 subunits with the exception of Dync1i1 (intermediate chain 1) (Fig 1A), which is found almost exclusively in the nervous system. To investigate the usage of light intermediate and light chains during IS formation, we expressed GFP-labeled forms of each subunit together with TagRFP-T-tubulin, a marker for the centrosome, in 5C.C7 effector T cells. All labeled subunits exhibited some degree of localization to cytoplasmic compartments surrounding the centrosome (S1 Fig), which was not surprising considering that dynein mediates the clustering of the Golgi apparatus and other organelles in this part of the cell [18]. The TCR photoactivation approach, however, enabled us to monitor a separate pool of dynein, which accumulates within the region of TCR stimulation before the centrosome polarizes and is likely responsible for its movement (white arrowheads in Fig 1B) [6, 8]. Both of the light intermediate chains of dynein 1 (Dync1li1 and Dync1li2), the roadblock light chains (Dylrb1 and Dylrb2), and the TcTex light chains (Dylt1b and Dylt3) exhibited this behavior, accumulating in the irradiated region just prior to centrosome reorientation toward the same zone. Interestingly, the LC8 light chains did not precede the centrosome in this manner, even though they did localize to pericentrosomal compartments and were therefore presumably assembled into dynein complexes. These results suggest that the pool of dynein 1 responsible for centrosome reorientation to the IS does not contain LC8 light chains but is otherwise unrestricted in its composition.

Although mature T cells do not possess primary cilia, they do express dynein 2 (Fig 1A). However, we did not observe recruitment of the dynein 2 light intermediate chain (Dync2li1) to the IS upon photoactivation (Fig 1B), suggesting that this motor complex is not involved in centrosome polarization in T cells. We also characterized the localization of two dynein cofactors, dynactin and Lis1. Dynactin, which binds to dynein via its largest subunit p150^Glued^, regulates dynein’s motor processivity and its recruitment to microtubule plus ends [19]. Lis1, for its part, directly binds to the motor domain of dynein and regulates its ability to move heavy cargoes [20]. To assess whether these cofactors are associated with synaptic dynein 1 during polarization responses, we tagged Lis1 and the dynamitin (p50) subunit of dynactin with GFP and imaged them together with the centrosome in photoactivation experiments. Both Lis1 and dynactin were recruited to the irradiated region prior to centrosome reorientation (Fig 1C), similar to the behavior we observed for dynein 1. Taken together, these results establish the relevant composition of dynein 1 complexes at the IS during TCR-induced polarization.

### TCR-induced F-actin clearance is required for dynein accumulation and centrosome reorientation

**Fig 2:**
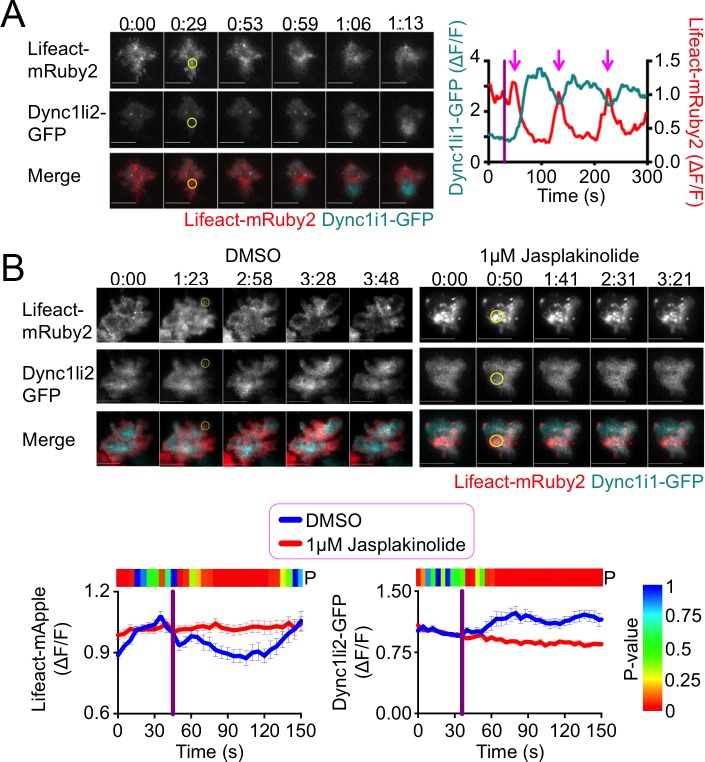
TCR-induced dynein accumulation requires F-actin depletion. TCR-induced dynein and F-actin dynamics in the presence or absence of 1 μM jasplakinolide were monitored by TCR photoactivation of 5C.C7 T cell blasts expressing Dync1li2-GFP and either Lifeact-mRuby2 or Lifeact-mApple. (A) A representative time-lapse montage is shown on the left, with the corresponding Lifeact-mRuby2 (red) and Dync1li2 (cyan) responses graphed to the right. Magenta arrows mark the cycles of F-actin depletion and dynein accumulation exhibited by this cell. (B) Top, representative time-lapse montages showing TIRF images of F-actin (left) and dynein (right) in the presence of 1 μM jasplakinolide or vehicle control (DMSO). Bottom, graphs showing mean actin depletion (left) and dynein accumulation (right) in the presence of 1 μM jasplakinolide or vehicle control (DMSO). The color bar above each graph indicates the P-value for each time point (two-tailed Student’s T-test). N ≥ 11 cells for each condition. In all time-lapse montages, the moment and location of UV irradiation is indicated by a yellow circle. Time in M:SS is indicated above each montage. Scale bars = 10 μm. In graphs, error bars denote SEM, and vertical purple lines indicate the moment of UV irradiation.

**Fig 3:**
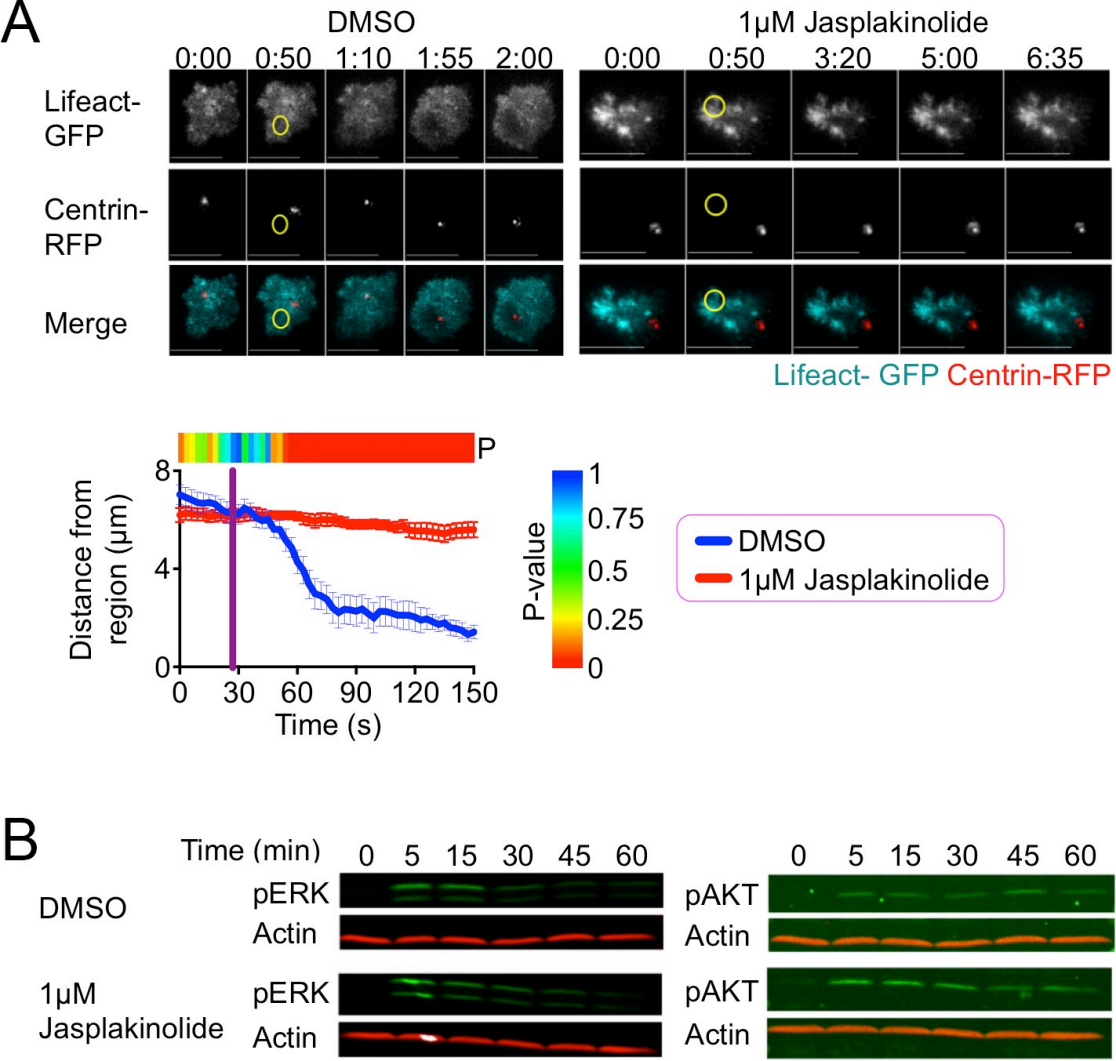
F-actin clearance promotes centrosome reorientation. (A) TCR photoactivation experiments were performed using 5C.C7 T cell blasts expressing Lifeact-GFP together with Centrin-TagRFP-T (Centrin-RFP) in the presence of 1 μM jasplakinolide or vehicle control (DMSO). Top, representative time-lapse montages showing TIRF images of F-actin and epifluorescence images of the centrosome. The moment and location of UV irradiation is indicated by a yellow circle. Time in M:SS is indicated above each montage. Scale bars = 10 μm. Bottom, centrosome reorientation was quantified as the mean distance between the centrosome and the center of the irradiated region over time. Error bars denote SEM, and the vertical purple line indicates the moment of UV irradiation. The color bar above the graph indicates the P-value for each time point (two-tailed Student’s T-test). N ≥ 11 cells for each condition. (B) 5C.C7 T cell blasts were stimulated using beads coated with MCC-I-E^k^ and ICAM-1 in the presence of 1 μM jasplakinolide or vehicle control (DMSO). pERK1/2 and pAKT responses were assessed at the indicated times by immunoblot.

Having characterized the composition and dynamics of the synaptic dynein complex, we next investigated potential mechanisms for dynein recruitment. Previous studies have documented a correlation between F-actin clearance at the IS and centrosome polarization [6, 13, 21]. To investigate whether F-actin dynamics could modulate dynein accumulation, we performed TCR photoactivation experiments using 5C.C7 T cells expressing the F-actin probe Lifeact (fluorescently labeled with either mRuby2 or mApple) together with Dync1li2-GFP (to monitor dynein). Accumulation of dynein in the irradiated region was markedly associated with the depletion of cortical F-actin (Fig 2A). This anti-correlation was particularly striking in cells that exhibited multiple cycles of Lifeact-mRuby2 depletion and recovery; in each cycle, the Dync1li2-GFP probe displayed inverse behavior (magenta arrows in Fig 2A). These results suggested a link between cortical F-actin and dynein recruitment at the IS. To explore this relationship, we carried out photoactivation experiments in the presence of jasplakinolide, a small molecule that prevents F-actin depolymerization and thereby stabilizes F-actin architecture. Jasplakinolide inhibited both Lifeact clearance as well as dynein accumulation at the irradiated region, consistent with the idea that F-actin depletion is a requisite step for the recruitment of dynein (Fig 2B). To probe the downstream consequences of this disruption, we examined centrosome reorientation in jasplakinolide-treated cells. Centrosome recruitment to the irradiated region was strongly inhibited by jasplakinolide (Fig 3A), indicating that F-actin clearance is necessary for T cell polarization.

Although these results were consistent with a specific role for F-actin dynamics in the recruitment of dynein, we also considered the possibility that jasplakinolide altered dynein accumulation secondarily by globally inhibiting TCR signaling. 5C.C7 T cells were preincubated with jasplakinolide or vehicle and then mixed with polystyrene beads coated with MCC-I-E^k^ and the adhesion protein ICAM-1, a ligand for the α_L_β_2_ integrin LFA-1. TCR-induced activation of the MAP kinase and phosphoinositide 3-kinase pathways was then assessed by immunoblot for phospho-Erk1/2 and phospho-AKT, respectively. Jasplakinolide did not alter these signaling outputs (Fig 3B), implying that its effects on dynein recruitment and centrosome reorientation reflected a specific role for F-actin dynamics in both of these processes.

### Localized PKC activity is required for F-actin clearance and dynein recruitment

**Fig 4:**
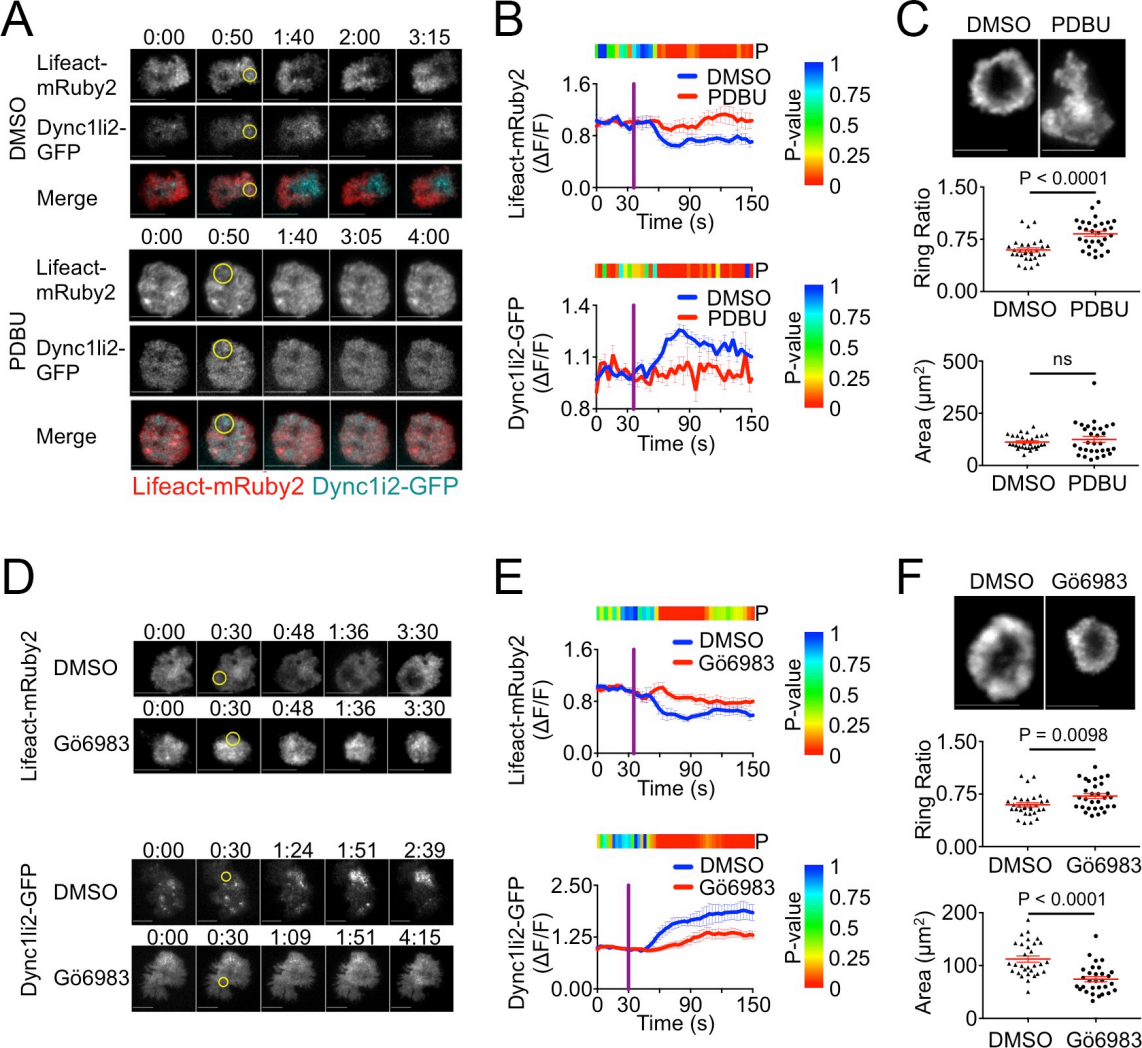
Polarized DAG and PKC signaling control synaptic F-actin clearance. (A-B) TCR photoactivation experiments were performed using 5C.C7 T cell blasts expressing Lifeact-mRuby2 together with Dync1li2-GFP in the presence of 1 μM PDBU or vehicle control (DMSO). (A) Representative time-lapse montages showing TIRF images of F-actin and dynein. The moment and location of UV irradiation is indicated by a yellow circle. Time in M:SS is indicated above each montage. Scale bars = 10 μm. (B) Quantification of mean F-actin clearance (above) and dynein accumulation (below) over time. Error bars denote SEM, and vertical purple lines indicate the moment of UV irradiation. The color bar above each graph indicates the P-value for each time point (two-tailed Student’s T-test). N ≥ 13 cells for each condition. (C) 5C.C7 T cell blasts were stimulated on supported lipid bilayers containing MCC-I-E^k^ and ICAM-1 in the presence of 1 μM PDBU or vehicle (DMSO), fixed, and stained with phalloidin to visualize F-actin. Above, TIRF images of representative synapses. Below, quantification of ring ratio (middle) and IS area (bottom). Mean values and error bars (SEM) are shown in red. N = 30 cells for each condition. (D-E) TCR photoactivation experiments were performed using 5C.C7 T cell blasts expressing Lifeact-mRuby2 together with Dync1li2-GFP in the presence of 50 nM Gö6983 or vehicle control (DMSO). (D) Representative time-lapse montages showing TIRF images of F-actin and dynein. Time in M:SS is indicated above each montage. Scale bars = 10 μm. (E) Quantification of mean F-actin clearance (above) and dynein accumulation (below) over time. Error bars denote SEM, and vertical purple lines indicate the moment of UV irradiation. The color bar above each graph indicates the P-value for each time point (two-tailed Student’s T-test). N ≥ 6 cells for each condition. (F) 5C.C7 T cell blasts were stimulated on supported lipid bilayers containing MCC-I-E^k^ and ICAM-1 in the presence of 50 nM Gö6983 or vehicle (DMSO), fixed, and stained with phalloidin to visualize F-actin. Above, TIRF images of representative synapses. Below, quantification of ring ratio (middle) and IS area (bottom). Mean values and error bars (SEM) are shown in red. N = 30 cells for each condition. P-values calculated from two-tailed unpaired Student’s T-test.

Previous work has established the importance of polarized DAG signaling for centrosome reorientation and dynein recruitment [8]. Accordingly, we investigated whether DAG accumulation is involved in F-actin depletion at the IS. To disrupt the effects of polarized DAG, we treated 5C.C7 T cells expressing Lifeact-mRuby2 and Dync1li2-GFP with phorbol 12,13-dibutyrate (PDBU), a phorbol ester that induces unpolarized DAG signaling. In photoactivation experiments, PDBU treatment disrupted both F-actin clearance and dynein accumulation at the irradiated region, indicating that polarized DAG is indeed necessary for both processes (Fig 4A and 4B).

To further assess DAG-mediated F-actin clearance at the IS, we imaged PDBU-treated 5C.C7 T cells on supported lipid bilayers coated with stimulatory MCC-I-E^K^ and ICAM-1. T cells typically form radially symmetric synapses on these bilayers containing a peripheral F-actin ring and a central domain that is depleted of F-actin. In the presence of PDBU, however, T cells displayed asymmetric patterns without obvious F-actin clearance in the center (Fig 4C). To quantify this organizational defect, we used the “ring ratio” parameter, which compares the fluorescence intensity at the periphery of the IS with that of the center [12]. A ring ratio less than one is indicative of an annular fluorescence pattern, whereas a ring ratio of one denotes a uniform distribution. PDBU treatment led to a substantial increase in ring ratio (Fig 4C), consistent with profound disruption of F-actin clearance. Despite these dramatic effects on F-actin configuration, PDBU did not significantly alter IS size (Fig 4C). Hence, the polarized delivery of DAG signals is crucial for IS organization, but it appears to be unnecessary for the formation of the contact itself.

DAG drives centrosome reorientation and dynein recruitment to the IS at least in part through PKCs [10]. To explore the role of these proteins in F-actin clearance, we performed photoactivation experiments using 5C.C7 T cells pretreated with the PKC inhibitor Gö6983. F-actin clearance and dynein accumulation at the irradiated region were both attenuated by Gö6983 (Fig 4D and 4E), consistent with a role for PKC activity in both processes. Gö6983 also inhibited F-actin ring formation on stimulatory lipid bilayers (Fig 4F), although its effects were less dramatic than those of PDBU. Interestingly, Gö6983-treated T cells formed significantly smaller synapses than vehicle-treated controls (Fig 4F), demonstrating that insufficient PKC activity can stunt IS growth. Collectively, these results indicate that polarized DAG and PKC signaling control the growth and organization of F-actin structures at the IS.

### Calcium signaling is dispensable for TCR-induced F-actin clearance

**Fig 5:**
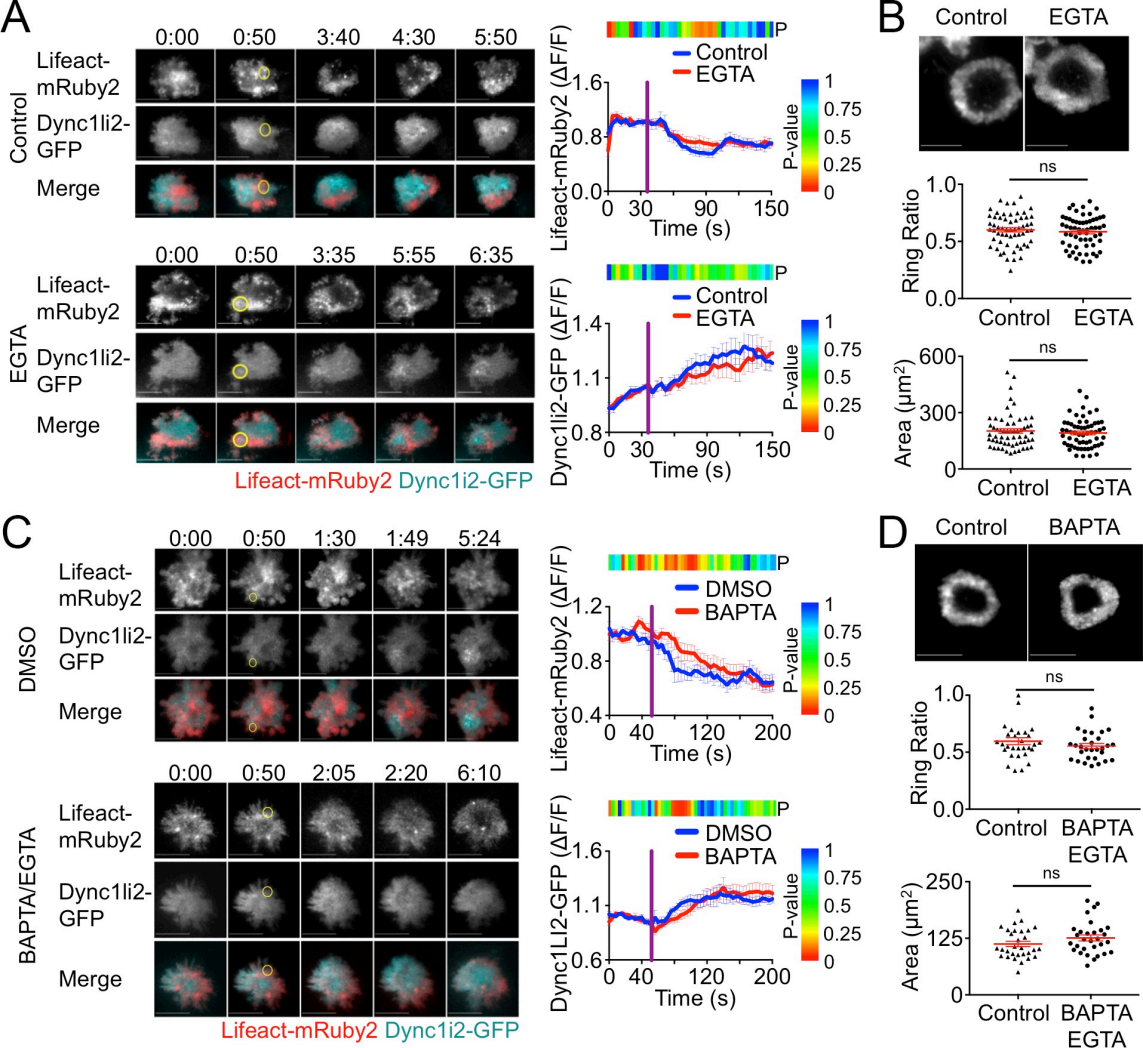
Calcium signaling is dispensable for F-actin clearance. (A, C) TCR photoactivation experiments were performed using 5C.C7 T cell blasts expressing Lifeact-mRuby2 together with Dync1li2-GFP in the presence or absence of 1 mM EGTA with 4 mM MgCl_2_ (A), or in the presence or absence of 1 mM EGTA and 50 μM BAPTA-AM with 4 mM MgCl_2_ (C). Left, representative time-lapse montages showing TIRF images of F-actin and dynein. The moment and location of UV irradiation is indicated by a yellow circle. Time in M:SS is indicated above each montage. Scale bars = 10 μm. Right, quantification of mean F-actin clearance (top) and dynein accumulation (bottom) over time. Error bars denote SEM, and vertical purple lines indicate the moment of UV irradiation. The color bar above each graph indicates the P-value for each time point (two-tailed Student’s T-test). N ≥ 14 cells for each condition. (B-D) 5C.C7 T cell blasts were stimulated on supported lipid bilayers containing MCC-I-E^k^ and ICAM-1 in the presence or absence of 1 mM EGTA with 4 mM MgCl_2_ (B), or in the presence or absence of 1 mM EGTA and 50 μM BAPTA-AM with 4 mM MgCl_2_ (D). The cells were then fixed and stained with phalloidin to visualize F-actin. Above, TIRF images of representative synapses. Below, quantification of ring ratio (middle) and IS area (bottom). Mean values and error bars (SEM) are shown in red. N ≥ 30 cells for each condition. P-values calculated from two-tailed unpaired Student’s T-test.

Previous work has suggested that calcium (Ca^2+^) flux is required for F-actin clearance at the IS [22]. To examine whether Ca^2+^ signaling controls F-actin depletion and dynein recruitment during centrosome polarization, we performed TCR photoactivation experiments using T cells expressing Lifeact-mRuby2 and Dync1li2-GFP in medium containing EGTA, a chelator that sequesters extracellular Ca^2+^. Surprisingly, EGTA had no effect on F-actin clearance from the irradiated region (Fig 5A). Dynein accumulation was also normal, reaffirming the close relationship between these two processes. To further investigate the role of Ca^2+^ in synaptic F-actin clearance, we incubated T cells on stimulatory lipid bilayers in the presence of EGTA and imaged the resulting synapses. We found that neither IS size nor F-actin clearance was affected by Ca^2+^ depletion (Fig 5B), in agreement with the photoactivation experiments.

Although EGTA treatment largely eliminates TCR-induced Ca^2+^ flux, it does not block Ca^2+^ release from the endoplasmic reticulum (ER), which can slightly increase cytoplasmic Ca^2+^ concentration. It was therefore possible that the synaptic F-actin clearance we observed was caused by Ca^2+^ release from ER stores. To address this alternative hypothesis, we performed additional experiments using a combination of EGTA and BAPTA-AM, and intracellular chelator that sequesters ER Ca^2+^ (Fig 5C and 5D). The results of these studies were indistinguishable from those performed using EGTA alone (Fig 5A and 5B), lending further support to the conclusion that Ca^2+^ signaling does not strongly affect synaptic F-actin architecture and dynein recruitment.

### Microtubules and dynein activity are dispensable for TCR-induced dynein recruitment

**Fig 6:**
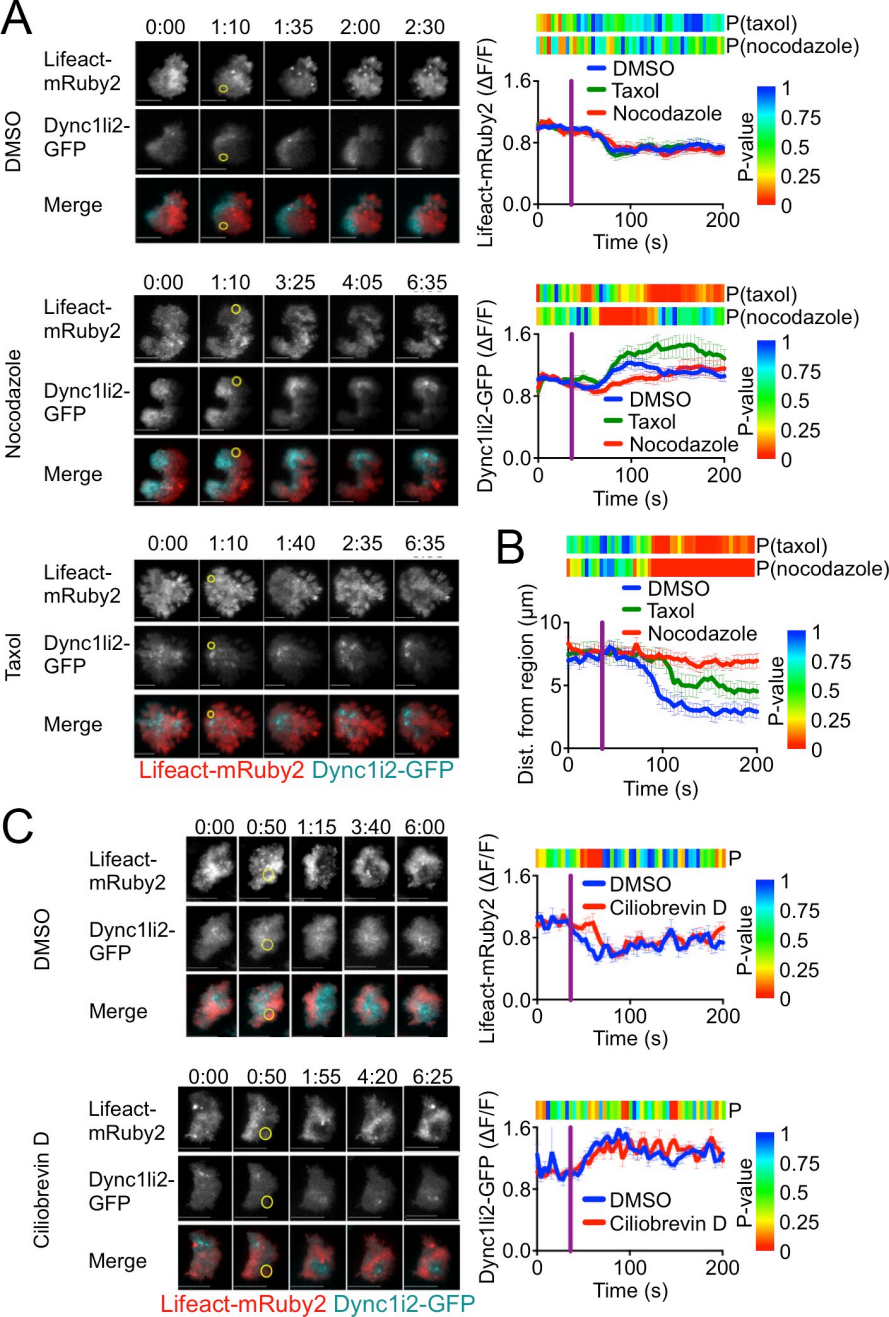
Microtubules and dynein activity are dispensable for F-actin depletion and dynein recruitment. (A) TCR photoactivation experiments were performed using 5C.C7 T cell blasts expressing Lifeact-mRuby2 together with Dync1li2-GFP in the presence of 30 μM nocodazole, 1 μM taxol, or vehicle control (DMSO). Left, representative time-lapse montages showing TIRF images of F-actin and dynein. Right, quantification of mean F-actin clearance (top) and dynein accumulation (bottom) over time. Color bars above each graph represent the P-value for each time point (two-tailed Student’s T-test), calculated by comparing the indicated experimental condition to the vehicle control. N ≥ 14 cells for each condition. (B) TCR photoactivation experiments were performed using 5C.C7 T cell blasts expressing Centrin-TagRFP-T in the presence of 30 μM nocodazole, 1 μM taxol, or vehicle control (DMSO). Centrosome reorientation was quantified by determining the mean distance between the centrosome and the center of the irradiated region over time. Color bars above the graph represent the P-value for each time point (two-tailed Student’s T-test), calculated by comparing the indicated experimental condition to the vehicle control. N ≥ 14 cells for each condition. (C) TCR photoactivation experiments were performed using 5C.C7 T cell blasts expressing Lifeact-mRuby2 together with Dync1li2-GFP in the presence of 50 μM ciliobrevin D or vehicle control (DMSO). Left, representative time-lapse montages showing TIRF images of F-actin and dynein. Right, quantification of mean F-actin clearance (top) and dynein accumulation (bottom) over time. The color bar above each graph indicates the P-value for each time point (two-tailed Student’s T-test). N ≥ 9 cells for each condition. In all time-lapse montages, the moment and location of UV irradiation is indicated by a yellow circle. Time in M:SS is indicated above each montage. Scale bars = 10 μm. In all graphs, error bars denote SEM, and vertical purple lines indicate the moment of UV irradiation.

Microtubules are thought to deliver dynein to the plasma membrane in other cell types [23, 24]. To examine whether a similar mechanism mediates dynein recruitment to the IS, we applied small molecule modulators of the microtubule cytoskeleton. 5C.C7 T cells expressing Lifeact-mRuby2 and Dync1li2-GFP were treated with either the microtubule depolymerizing agent nocodazole or the stabilizing agent taxol and then subjected to TCR photoactivation. Neither nocodazole nor taxol affected TCR-induced F-actin dynamics (Fig 6A). In both cases, clearance of Lifeact-mRuby2 from the irradiated region was essentially indistinguishable from that of control cells. Nocodazole-treated and taxol-treated T cells also exhibited robust dynein recruitment to the irradiated region (Fig 6A). Indeed, in the case of taxol, Dync1li2-GFP accumulation was consistently more intense than in cells treated with vehicle alone. Taken together, these results strongly suggest that microtubules are not involved in dynein delivery to the IS. Importantly, both nocodazole and taxol inhibited centrosome reorientation in photoactivation experiments (Fig 6B). Hence, while dynamic microtubules are required for cytoskeletal polarization, they operate at a step downstream of motor protein recruitment.

Finally, we examined whether the motor activity of dynein is necessary for its own recruitment. 5C.C7 T cells expressing Lifeact-mRuby2 and Dync1li2-GFP were subjected to TCR photoactivation in the presence of ciliobrevin D, a small molecule dynein inhibitor. TCR-induced F-actin clearance and dynein accumulation were unaffected by ciliobrevin D (Fig 6C), indicating that motor activity is not required for dynein accumulation at the IS.

## Discussion

Proper IS function relies not only upon robust F-actin polymerization at the cell-cell interface but also upon its targeted depletion. Cortical F-actin clearance within the IS plays a well-established role in the directional secretion of cytotoxic molecules toward the target cell [25, 26], and it has also been linked to the reorientation of the centrosome [13, 21]. Here, we demonstrate the importance of F-actin depletion for the synaptic accumulation of dynein, a microtubule motor that is critical for both T cell polarity and intracellular trafficking. This is consistent with the idea that F-actin and microtubule dynamics are closely coupled at the IS [27], which is fitting for a structure that mediates highly coordinated exo- and endocytosis.

Although our work highlights the importance of removing F-actin from IS, it should be noted that F-actin is never completely cleared from the synaptic membrane. Indeed, super-resolution imaging studies have revealed that a sparse network of fibers persists in the center of the IS even after centrosome reorientation [25, 26, 28]. The fact that this residual, hypodense F-actin network can coexist with synaptic dynein implies that F-actin density need only drop below a certain threshold to permit dynein recruitment. It is also possible, however, that dynein recruitment is not antagonized by F-actin per se, but rather by a specific type of F-actin structure. The dense, branched network that prevails at the leading edge of lammelipodia, for example, could be particularly inhibitory. The application of super-resolution imaging approaches should enable investigators to address this issue in greater depth.

The diversity of dynein isoforms, light chain subunits, and cofactors presumably facilitates functional specialization of the motor in different intracellular contexts. Our imaging experiments suggest that the synaptic pool of dynein involved in centrosome polarization contains the intermediate and light intermediate chains of cytoplasmic dynein1 along with the roadblock and Tctex light chains. Interestingly, we did not observe accumulation of LC8 in the region of TCR stimulation, although it did appear to decorate centrosome-associated compartments. These results imply a division of labor between the dynein light chains at the IS, with LC8 contributing to organelle accumulation at the centrosome but not centrosome reorientation per se. This interpretation is consistent with prior studies indicating that dynein light chains contribute differentially to certain cellular processes and that distinct light chain compositions imbue dynein holoenzymes with specialized functions [29, 30]. Notably, we did not observe synaptic recruitment of cytoplasmic dynein2, the isoform associated with intraflagellar transport at cilia [17]. Recent studies have highlighted the structural and molecular similarities between the IS and primary cilia [31, 32]. Dynein usage does not appear to be one of them, at least in the context of centrosome reorientation. We do not exclude the possibility, however, that dynein2 may contribute to other trafficking events within the mature IS, such as the coalescence of lytic granules or the centripetal movement of TCR signaling microclusters [33, 34]. We also observed accumulation of dynamitin and Lis1 in the region of TCR stimulation, implying that both the dynactin and the Lis1/NDE1 complexes are involved in IS assembly. The precise role of dynactin in T cell polarity is somewhat controversial. Although overexpression of the dynamitin subunit was initially shown to impair centrosome reorientation [5], recent shRNA knockdown studies have suggested that it is Lis1/NDE1, and not dynactin, that operates in this context [35]. Although our data are consistent with roles for both complexes, they do not exclude the possibility that dynactin may associate with the synaptic dynein complex without contributing to its function.

Blocking TCR-induced Ca^2+^ signaling had no effect on F-actin clearance and centrosome reorientation in our hands. These results, although consistent with our previous work [8], contradict a recent report indicating that Ca^2+^ is required for synaptic F-actin depletion [22]. Whereas we employed Lifeact to label F-actin in our live imaging experiments, the previous study used F-tractin, which is thought to label a larger fraction of filaments [36]. Hence, it is formally possible that we failed to observe a Ca^2+^ sensitive pool of F-actin at the IS that could be detected by F-tractin but not by Lifeact. Incomplete Lifeact labeling, however, does not explain our phalloidin staining experiments, which also failed to support a role for Ca^2+^ signaling. Furthermore, even if a Ca^2+^ sensitive pool of filaments exists, our other results imply that it does not influence dynein accumulation and centrosome reorientation. It is possible that the discrepancies in question reflect species-specific effects; we used primary murine T cells while the previous study used human material [22]. Recently, it was shown that human, but not mouse, NK cells require Ca^2+^ signaling for lytic granule exocytosis [37]. Hence, it is not implausible that synaptic F-actin dynamics might be governed by different signaling constraints in human and mouse T cells.

Previously, we showed that synaptic accumulation of DAG and nPKCs downstream of PLCγ is required for centrosome polarization [8, 10]. Our present work extends these observations by demonstrating that this same pathway also controls F-actin clearance and dynein accumulation. The nPKC substrates responsible for mediating these effects remain to be identified. Myristoylated alanine rich PKC substrate (Marcks) and the related Marcksl1 are intriguing candidates in this regard. Marcks family proteins are thought to couple the plasma membrane to the F-actin cytoskeleton [38]. PKC-mediated phosphorylation releases Marcks and Marcksl1 from the membrane and could therefore alter cortical F-actin architecture. We have found that TCR stimulation induces Marcksl1 depletion from the IS [10], and it will be interesting to investigate whether loss of one or both proteins disrupts F-actin clearance, dynein recruitment, and centrosome reorientation. PKC independent DAG signaling is also likely to play a role in these events. Indeed, the fact that PBDU (which mimics unpolarized DAG) disrupted synaptic F-actin architecture more strongly than did PKC inhibition (via Gö6983) in our hands implies that PKCs are necessary but not sufficient for T cell polarization. Furthermore, recent studies have implicated PIP_2_ depletion in synaptic F-actin clearance, highlighting the importance of PLCγ not only as a generator of DAG but also as a consumer of PIP_2_ [11, 39]. Deciphering the choreography of these diverse PLCγ dependent pathways will be an important step toward understanding the complex biology of the IS and of immune cell-cell interactions more generally.

## Materials and methods

### Ethics statement

The animal protocols used for this study were approved by the Institutional Animal Care and Use Committee of Memorial Sloan-Kettering Cancer Center.

### T cell culture and retroviral transduction

CD4^+^ T cells derived from transgenic mice expressing the 5C.C7 T-cell receptor were stimulated by coculture with B10A splenocytes at a ratio of 1:10 in RPMI medium containing 10% FBS and 5μM moth cytochrome c_88-103_ (MCC) peptide. 30 U/mL of IL-2 was added to cells 16 h following T cell activation. Subsequently, cells were split as needed into RPMI containing 30 U/mL of IL-2. T cells were retrovirally transduced with fluorescent probes as previously described 72 hours after the initiation of the T cell culture [8]. 24 hours post-transduction, cells were placed under puromycin selection (5 μg/mL). After an additional 48 h, transduced T cells were isolated by centrifugation over histopaque (Sigma).

### Signaling probes

Lifeact constructs have been described [6, 8]. The full-length coding sequence of centrin 2 was amplified from RNA derived from 5C.C7 T cell blasts and ligated into a murine stem cell virus (MSCV) retroviral expression vector upstream of and in-frame with Tag-RFP-T. Cytoplasmic dynein subunits were cloned from RNA isolated from 5C.C7 T cell blasts. All light intermediate chains (Dync1li1, Dync1li2 and Dync2li1), Tctex-1 light chain (Dynlt1), roadblock family light chains (Dynlrb1 and Dylrb2) and LC8 family light chains (Dynll1 and Dynll2) were inserted into pMSCV upstream and in-frame of GFP. Tctex-3 light chain was inserted into MSCV retroviral vector downstream and in-frame of GFP. For visualization of Lis1 and the Dynactin complex, we amplified full length Lis1 and the dynamitin (p50) subunit of Dynactin, respectively, from RNA isolated from 5C.C7 T cell blasts, and ligated them into pMSCV downstream and upstream of the fluorescent protein cassette, respectively.

### Photoactivation and TIRF imaging

T cells expressing probes of interest were transferred into minimal imaging medium lacking phenol red and then attached to glass coverslips coated with NPE-MCC-I-E^k^ (125 ng/ml), nonstimulatory I-E^k^ containing a peptide derived from hemoglobin (amino acids 64–76; 3 μg/ml) and an antibody against H-2K^k^ (to encourage T cell attachment to the surface; 0.5μg/ml, BD Biosciences) [8]. Time-lapse images were recorded every 5 s for a total of 7 minutes using an inverted fluorescence microscope (Olympus) fitted with a 150× objective (1.45 NA). Fluorescent probes were all visualized using TIRF illumination except the centrosomal probes (tubulin and centrin), which were imaged in epifluorescence mode. GFP and RFP/mRuby2/mApple excitation was achieved using 488 nm and 561 nm lasers respectively. T cells were photoactivated with a 1.5 s UV pulse after the 14^th^ time point. UV irradiation of defined regions was performed using a Mosaic digital diaphragm system (Photonic Instruments) attached to a mercury lamp (Olympus). Small-molecule inhibitors and pharmacological agents were added to the medium above the cells as 200x stocks in DMSO; final concentrations were 50 nM Gö6983 (BioVision), 1 μM PDBU (Cell Signaling), 1 μM jasplakinolide (EMD Millipore), 30 μM nocodazole (MedChem Express), 1 μM taxol (MedChem Express), 50 μM ciliobrevin D (EMD Millipore), and 1 mM EGTA with 4 mM MgCl_2_ or 1 mM EGTA and 50 μM BAPTA-AM with 4 mM MgCl_2_ for calcium blockade.

### Lipid bilayers and TIRF imaging

Supported lipid bilayers containing streptavidin and biotinylated proteins were prepared as previously described [12]. To activate 5C.C7 T cells, lipid bilayers were coated with 1 μg/ml of biotinylated MCC-I-E^k^ and 1 μg/ml ICAM-1. T cells were incubated on stimulatory bilayers for 15 min at 37°C, followed by fixation in 2% paraformaldehyde for 10–15 minutes at room temperature (RT). For phalloidin staining, fixed cells were permeabilized using 0.5% Triton X-100 for 5 min, followed by incubation with PBS solution. Cells were then incubated with 1 μg/mL Alexa Fluor 594-labeled phalloidin in PBS for 1–2 hours at RT. T cells were imaged by TIRF microscopy with a 60× objective lens using a 561 nm laser. Small molecule inhibitors and pharmacological reagents were mixed with the T cells prior to their addition to the lipid bilayers; final concentrations were 50 nM Gö6983 (BioVision), 1 μM PDBU (Cell Signaling) and 1 mM EGTA with 4 mM MgCl_2_ or 1 mM EGTA and 50 μM BAPTA-AM with 4 mM MgCl_2_ for calcium blockade.

### Image analysis

For photoactivation experiments, images were analyzed using SlideBook (Intelligent Imaging Innovations) and Microsoft Excel and graphed using Prism (GraphPad). Centrosome polarization was quantified by measuring the distance between the centrosome and the center of the UV irradiated region at each time point. Depletion or accumulation of fluorescent signaling probes at the irradiated region was quantified by calculating the mean fluorescence intensity (MFI) within the region for each time point. The MFI was normalized using the first ten time points prior to photoactivation, following background correction. T cell spreading on lipid bilayers was determined by calculating the area of the Alexa Fluor 594-labeled phalloidin signal. To calculate the ring ratio, the background-corrected mean fluorescence intensity (MFI) was compared to the MFI at center of the IS as previously described [12]. Ring ratios calculated from two perpendicular line scans were averaged to yield a single value for each cell.

### Immunoblot analysis

For T cell activation, 5C.C7 T cells were preincubated with 1 μM jasplakinolide or DMSO for 15 minutes at 37°C. Following treatment, T cells were mixed with polystyrene beads coated with 1 μg/mL of MCC-I-E^k^ and 1 μg/mL ICAM-1 and incubated at 37°C. At various time points, cells were collected and lysed in cold lysis buffer containing 50 mM TrisHCl, 0.15 M NaCl, 1 mM EDTA, 1% NP-40, 0.25% sodium deoxycholate, phosphatase inhibitors (1 mM NaF and 0.1 mM Na_3_VO_4_), and protease inhibitors (cOmplete mini cocktail, EDTA-free, Roche). Activation of PI3K and MAP kinase signaling was assessed by immunoblot for pAkt (Phospho-Akt (Ser473) Ab; Cell Signaling Technology) and pErk1/2 (Phospho-Thr202/ Tyr204; clone D13.14.4E; Cell Signaling Technology), respectively.

## Supporting information

S1 Fig Labeled dynein chains accumulate in pericentrosomal compartments. 5C.C7 T cell blasts expressing fluorescently labeled tubulin (to visualize the centrosome) together with the indicated GFP-labeled dynein light intermediate and light chains were used in TCR photoactivation experiments. Images show photoactivated cells prior to centrosome reorientation, with the irradiated region denoted by a yellow circle. In each panel, a TIRF image of the labeled dynein chain together with an epifluorescence image of RFP-tubulin is shown above, with the same dynein image shown on its own below. White arrows indicate the position of the centrosome. Scale bars = 10 μm.
(TIF)

S1 Table Raw data for Fig 1B and 1C. (XLSX)

S2 Table Raw data for Fig 2. (XLSX)

S3 Table Raw data for Fig 3A. (XLSX)

S4 Table Raw data for Fig 4. (XLSX)

S5 Table Raw data for Fig 5. (XLSX)

S6 Table Raw data for Fig 6. (XLSX)
