# Supplementary figures and images for: Actin clearance promotes polarized dynein accumulation at the immunological synapse
Source: PLoS One. 2019 Jul 3;14(7):e0210377. doi: 10.1371/journal.pone.0210377 (PMC6608937; doi:10.1371/journal.pone.0210377)

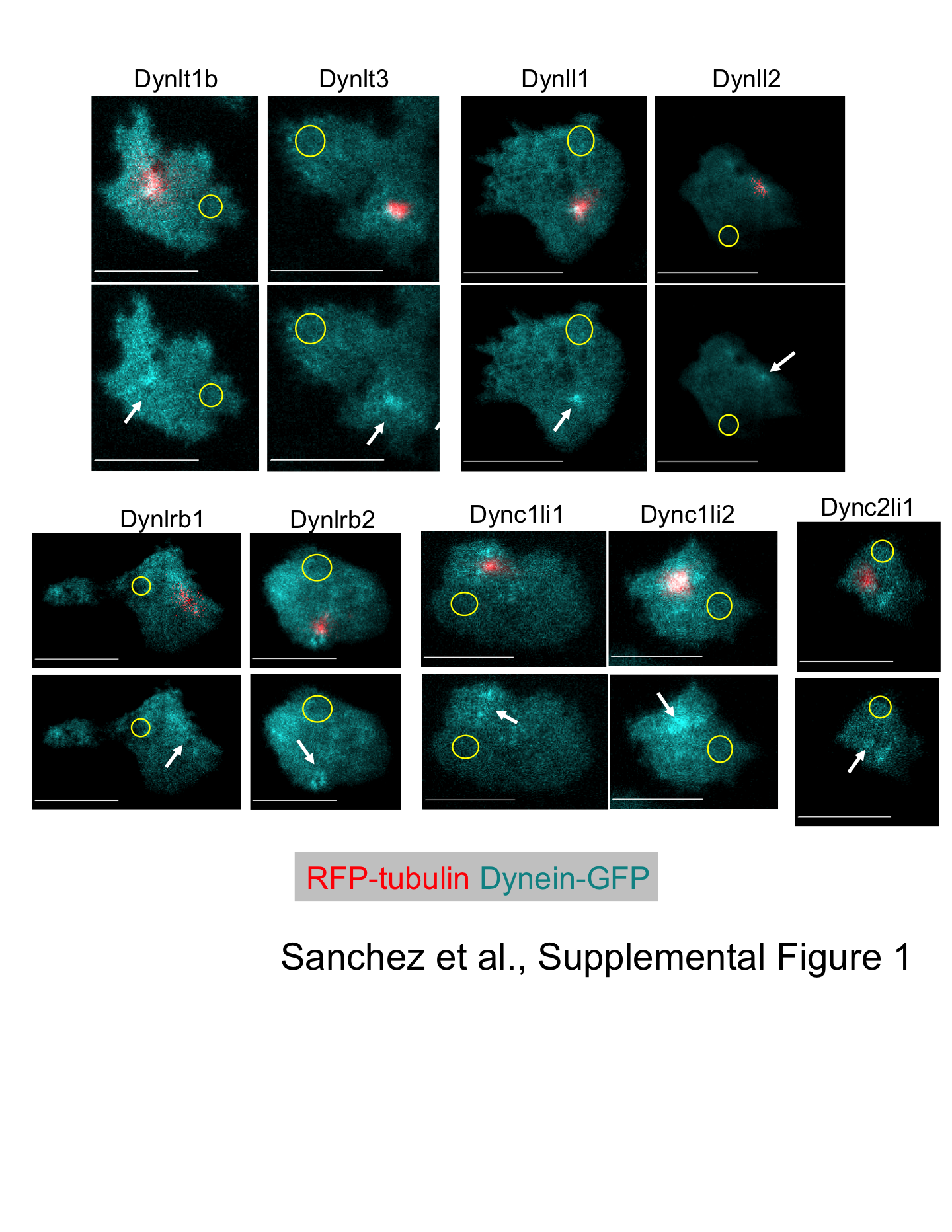

Supplement: S1 Fig — 5C.C7 T cell blasts expressing fluorescently labeled tubulin (to visualize the centrosome) together with the indicated GFP-labeled dynein light intermediate and light chains were used in TCR photoactivation experiments. Images show photoactivated cells prior to centrosome reorientation, with the irradiated region denoted by a yellow circle. In each panel, a TIRF image of the labeled dynein chain together with an epifluorescence image of RFP-tubulin is shown above, with the same dynein image shown on its own below. White arrows indicate the position of the centrosome. Scale bars = 10 μm. (TIF) [file pone.0210377.s001.tif]
